# Near Infrared Spectroscopy Study of the Frontopolar Hemodynamic Response and Depressive Mood in Children with Major Depressive Disorder: A Pilot Study

**DOI:** 10.1371/journal.pone.0086290

**Published:** 2014-01-23

**Authors:** Masahide Usami, Yoshitaka Iwadare, Masaki Kodaira, Kyota Watanabe, Kazuhiko Saito

**Affiliations:** 1 Department of Child and Adolescent Psychiatry, National Center for Global Health and Medicine, Kohnodai Hospital, Ichikawa, Japan; 2 Department of Child Mental Health, Imperial Gift Foundation, Aiiku Hospital, Tokyo, Japan; Rikagaku Kenkyūsho Brain Science Institute, Japan

## Abstract

**AIM:**

The aim of this study was to evaluate the frontopolar hemodynamic response and depressive mood in children with mild or moderate major depressive disorder during six weeks treatment without medication.

**METHODS:**

The subjects were 10 patients with mild or moderate depression. They were depressive drug-naive children and adolescents. The scores of Depression Self Rating Scale (DSRS), the results of the Verbal Fluency Test (VFT), and the concentrations of oxy-hemoglobin (Oxy-Hb) of frontal pole brain assessed by two-channel near infrared spectroscopy (NIRS) after six weeks of treatment was compared with those of initial treatment.

**RESULTS:**

The score of DSRS was significantly reduced after six weeks of initial treatment (p<0.001, t-test). The word number of VFT was not significantly changed after six weeks of treatment. The oxy-Hb concentration significantly increased after six weeks of treatment (p<0.001, t-test).

**CONCLUSIONS:**

This study demonstrated that the concentration of oxy-Hb of frontopolar cortex in children with mild and moderate depression improved along with their depressive mood. These results suggested that concentration of oxy-Hb using NIRS may be used as the state maker for change in depressive mood of children having depression, similar to that in adults.

## Introduction

Treating children and adolescents with major depressive disorder (depression) or clinical depression has some limitations. These limitations include difficulty in diagnosis of childhood depression and safety and efficacy of pharmacotherapy for depression among children and adolescents [1]–[4].

For diagnosis of depression, the use of diagnostic criteria is essential even in children and adults. However, children find it difficult to communicate their feelings, such as depressive mood [5]. Furthermore, children with depression do not want to talk, tend to be irritable (grumpy, hostile, easily frustrated, angry outbursts), and have extreme sensitivity to rejection [6]. Furthermore, in the absence of clear-cut biomarkers for depression, clinicians depend on a subjective examination [7]. Therefore, the diagnosis of children with depression is a very difficult issue in clinical practice. Some guidelines recommend 4–6 weeks of psychosocial treatment for mild and moderate depression among children and adolescents rather than pharmacotherapy [6], [8], [9]. Children with severe depression require hospitalization, and in cases of suicidal attempts the child must be treated with pharmacotherapy [8]–[10]. The use of antidepressants such as selective Sserotonin reuptake inhibitors (SSRIs), for children and adolescents with depression has been discussed as an effective and safe form of treatment [10]–[16]. Therefore, it is necessary to evaluate their depressive state, the need for pharmacotherapy, and the efficacy of pharmacotherapy using some questionnaires such as the Depression Self-Rating Scale (DSRS), the Child Behavior Checklist [17], the Strength and Difficulties Questionnaire [18], [19], and the Children with Difficulties Questionnaire [20]–[23].

Recently, developments in near infrared spectroscopy (NIRS) have enabled noninvasive examination of brain function [24], [25]. Combined with other diagnostic techniques, NIRS could be a powerful tool. NIRS is used to measure the concentration of oxygenated hemoglobin (oxy-Hb) and deoxygenated hemoglobin concentration in the capillaries. Based on the hypothesis that hematocrit is constant, it is possible to estimate the changes in cerebral blood flow with this technique [25]. The technique takes advantage of the fact that compared with constituents of other tissues, hemoglobin in blood absorbs more light in NIRS. Blood flow to a particular brain region increases when neurons are active. Thus, monitoring the changes in hemoglobin concentration gives a site-specific read on blood flow and in turn on neuronal activity [7], [26]. Change in the frontopolar hemodynamic response was evidenced in a previous study on adult parents with depression [27]. NIRS offers an objective measure of mental health, which is reliable and convenient for routine use in the clinic; therefore, the technique was approved by the health ministry as an “advanced medical technology” to assist psychiatric diagnoses in 2009 [7] [28].

There are a few studies of children using NIRS [29]–[33]; however, these studies focused on children with attention deficit/hyperactivity disorder and pervasive developmental disorder [32], [33]. There are no studies using NIRS for depression in children and adolescents. There is a need to evaluate children with depression using NIRS. Therefore, it is important to discuss about how children and adolescents with depression have reduced blood flow in the prefrontal cortex. The aim of this study was to evaluate the relationship between the change in the frontopolar hemodynamic response and the change in depressive mood in children with mild and moderate depression during the first six weeks of treatment. We used NIRS to evaluate the frontopolar hemodynamic response during the Verbal Fluency Test (VFT). VFT has been used widely in patients along with NIRS to evaluate depressive states [24], [25], [27], [34]–[36].

This study was a pilot study using two-channel NIRS. The main hypothesis is that the activity of the frontopolar cortex and depressive mood improved among children and adolescents with depression during six-week treatment without use of medication. Therefore, the frontopolar hemodynamic response is considered a state maker for the depressive mood of a child with depression.

## Methods

Along with the subjects’ documented informed assent, the subject’s parents also gave written informed consent for participation in the study. The ethics committee of the National Center for Global Health and Medicine approved this study.

### Participants

This study was a prospective study. A total of 10 individuals [1 boy, 9 girls; age: 12.9±0.9 years (mean ± standard deviation), 12–15 years, range] were examined. They were diagnosed as having mild or moderate depression according to DSM-IV-TR criteria by a psychiatrist, who is a specialist in child and adolescent psychiatry at the department of child and adolescent psychiatry, Kohnodai Hospital, National Center for Global Health and Medicine between July 1st, 2009 and December 31, 2011. The Kohnodai Hospital is located in Ichikawa City, in the eastern part of the Tokyo metropolitan area. We have no real data regarding the socioeconomic status of the citizens of Ichikawa City. Ichikawa City has fully developed into a residential area and center for education.

This study excluded students with coexisting mental disorders including mental retardation, pervasive developmental disorders, eating disorders, attention deficit/hyperactivity disorders, or oppositional defiant disorders. They were not treated with pharmacotherapy, cognitive behavior therapy, or family therapy. We treated these children with psychodynamic therapy. These children had no history of suicidal attempts or ideation.

### Clinical assessment

Depressive symptoms and level of social functioning were evaluated by a child and adolescent psychiatrist using DSM-IV-TR criteria, the Depression Self-Rating Scale (DSRS) [20], [21], and Global Assessment of Functioning scores.

### Measures

#### Depression Self-Rating Scale (DSRS)

The DSRS is an 18-item measure of depressive symptoms for children [20]. Murata et al. [21] standardized the Japanese version of DSRS. The DSRS comprises 18 questions with the following options for answers: “always,” “sometimes,” or “never.” For questions related to depression, the responses of “always,” “sometimes,” and “never” are given 2, 1, and 0 points, respectively. Half of the 18 questions are reverse questions that have negative correlations with depression, and “always,” “sometimes,” and “never” in these items are given 0, 1, and 2 points, respectively. The total score is the sum of the 18 item scores. The total scale ranges from 0 to 27, with higher scores indicating a greater severity of depressive symptoms. According to Murata et al., the cut-off of total points for depression in the DSRS in Japan is 16.

#### Near infrared spectroscopy: NIRS

The NIR spectroscope that we used has two probes. The distance between a pair of source-detector probes was set at 3.0 cm and each area measured between a pair of source-detector probes was defined as a “channel.” The NIRS device is considered to measure “channels” at 2–3 cm depth from the scalp, that is, at the surface of the cerebral cortex [23], [24], [26], [33]–[36]. Many studies and an “advanced medical technology” in Japan used the multi-channel NIRS made by Hitach Medical Corporation or Shimadzu. This study used the two-channel NIRS made by Spectratech Inc. The two-channel NIRS is cheaper than the multi-channel NIRS, and the former can be attached to a patient more easily.

Since there are only two channels to efficiently measure the function of the prefrontal cortex, measurement positions were determined by the following method. In accordance with the international 10–20 system used in electroencephalography, two probes were positioned at Fp1 and Fp2. The activity of the frontopolar cortex decreased in adult patients having depression compared with that of normal controls [26]. The position of the probe of the two-channel NIRS can be freely adjusted with velcro. It is possible to match the Fp1 and Fp2 reliably.

The VFT method and measurement principle of NIRS were repeatedly explained to the patient using a brochure and demonstration. Patients were instructed to minimize motions such as head movements, strong biting, and blinking during NIRS measurement, to avoid artifacts.

The patients sat on a comfortable chair in a silent and day-lit room. Data clearly containing motion artifacts, based on both our observations and the NIRS recording, were excluded from further analyses.

#### Activation Task: the Verbal Fluency Test (VFT)

There are many studies using VFT in adults with depression [23], [24], [26], [33]–[36]. In clinical practice, depressive patients are widely evaluated using VFT and NIRS. It has been suggested that a decrease occurs in the concentration of oxy-Hb of the prefrontal cortex during VFT. We performed VFT according to the protocol established by Takizawa et al. [37]

VFT has been enforced in the following way. For a resting baseline condition, the subject was instructed to relax with eyes closed. For approximately 20 s, the subjects were asked to simply repeat Japanese vowels out loud “A,” “I,” “U,” “E,” and “O.” The letter version of the VFT was conducted. The verbal fluency task consisted of three different letter tasks that required the participants to pronounce as many nouns as possible beginning with a particular letter (first “Ha,” then “Ki” and “Fu”; no proper nouns, no repetitions). Each of the three task conditions lasted about 60 s. The correct verbal responses were recorded and used as a measure of behavioral performance. The duration of the baseline and the different task conditions were marked on the NIR spectroscope by the investigator.

### Statistical Analysis

Results were expressed as mean ± standard deviation (SD) of DSRS, word number of VFT, concentration of oxy-Hb, and GAF. These results were statistically compared by the t-test between initial treatment and after six weeks. The effect sizes were calculated based on the t-test statistics. The changing hemodynamic activity was calculated by subtracting the concentration of oxy-Hb after six weeks treatment from the concentration of oxy-Hb at initial treatment. We recorded the concentration of oxy-Hb in 120 s under the NIRS task. We calculated the average concentration of oxy-Hb every 0.1 s and compared the average concentration of oxy-Hb at initial treatment and after six weeks treatment.

In all tests, a significance level of 0.05 was used in two-sided tests. Analyses were performed using PASW 18.0 statistical software.

## Results

### Descriptive information

The participants included 10 children who were diagnosed with mild/moderate depression according to diagnosis criteria in the DSM-IV-TR. Table 1 shows the gender, age, GAF, serenity, and medication of 10 children.

**Table 1:** Gender, Age IQ, words number of VFT, DSRS score, and GAF score of 10 children with major depressive disorder.

				words number of VFT						DSRS score		GAF score	
Patients	Gender	Age	FIQ	Initial treatment			After six weeks			Initial treatment	After six weeks	Initial treatment	After six weeks
A	Girl	14	92	3	4	4	3	4	4	24	24	40	45
B	Boy	14	83	3	3	4	3	3	3	22	15	42	48
C	Girl	10	94	3	4	4	4	3	5	23	26	28	35
D	Girl	12	92	4	2	3	4	4	4	26	20	33	38
E	Girl	14	101	3	2	3	3	3	4	18	17	48	50
F	Girl	13	89	3	4	4	3	5	4	16	16	48	50
G	Girl	10	99	2	4	4	2	4	5	24	18	47	50
I	Girl	13	115	4	4	3	4	4	4	26	23	37	34
J	Boy	14	103	3	3	4	3	3	4	21	23	40	38
K	Girl	15	95	2	2	4	3	3	4	26	19	38	45
Mean	-	12.9	96.3	3	3.2	3.7	3.2	3.6	4.1	22.4	19.7	40.1	43.1
SD	-	1.7	8.8	0.7	0.9	0.5	0.6	0.7	0.6	3.61	3.67	6.95	6.81

IQ, intelligence quotient; VFT, Verbal Fluency Test; DSRS score, Depression Self Rating Scale; and GAF score, Global Assessment of Functioning.

### Change of DSRS during six weeks treatment

Table 1 showed the score of DSRS of each participant at initial treatment and after six weeks. The score of DSRS at initial treatment and at after six weeks was 22.4±3.6 and 19.7±3.6, respectively. The mean score of DSRS at after six weeks was significantly lower than that of DSRS at initial treatment (Table 1, p<0.001, t-test). The effect size was 0.75.

### VFT and NIRS

The results of VFT at initial treatment and after six weeks were compared. The mean word number of VFT after six weeks was not significantly different between initial treatment and after six weeks (N.S., t-test).

**Figure 1:**
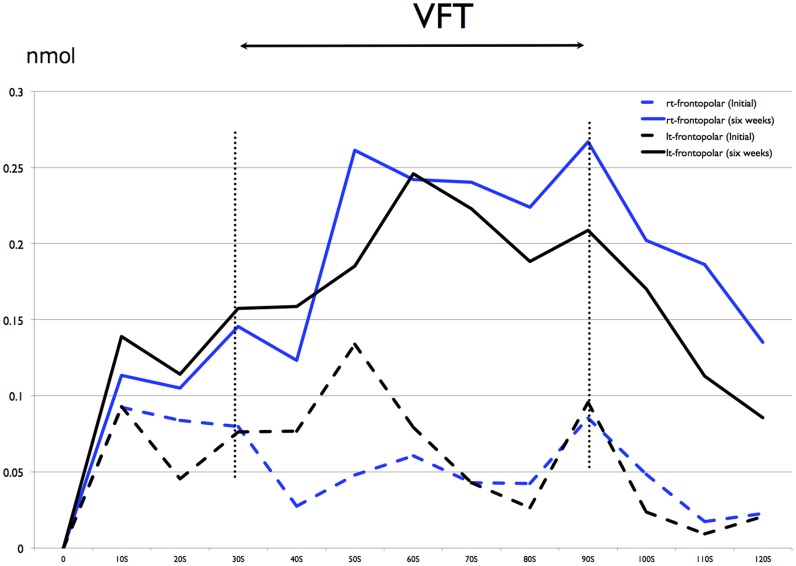
Concentration of oxy-Hb of frontopolar cortex during VFT at initial treatment and after six weeks.

Figure 1 shows the concentration of oxy-Hb at initial treatment and after six weeks. Average concentrations of oxy-Hb at initial treatment and after six weeks were compared

Before the letter version of the VFT (0–29 s), the concentration of oxy-Hb at initial treatment was 0.05±0.07 nmol (rt) and 0.05±0.07 nmol (lt), the concentration of oxy-Hb at after six weeks treatment was 0.06±0.08 nmol (rt) and 0.07±0.10 nmol (lt). The concentration of oxy-Hb was no significantly changed than the concentration of oxy-Hb at after six weeks (t-test).

Under the letter version of the VFT (30–89 s), the concentration of oxy-Hb at initial treatment was 0.06±0.04 nmol (rt) and 0.07±0.03 nmol (lt), the concentration of oxy-Hb at after six weeks treatment was 0.20±0.22 nmol (rt) and 0.18±0.19 nmol (lt). The concentration of oxy-Hb was significantly lower than the concentration of oxy-Hb at after six weeks (p<0.001, t-test). These effect sizes were 1.67 (right frontopolar) and 1.66 (left frontopolar).

After the letter version of the VFT (90–120 s), the concentration of oxy-Hb at initial treatment was 0.02±0.00 nmol (rt) and 0.01±0.01 nmo (lt), the concentration of oxy-Hb at after six weeks treatment was 0.16±0.04 nmol (rt) and 0.10±0.02 nmol (lt). The concentration of oxy-Hb was no significantly changed than the concentration of oxy-Hb at after six weeks (t-test).

## Discussion

This study showed that the activity of the frontopolar cortex and depressive mood was improved among children and adolescents with depression after six weeks treatment. These results suggested that the frontopolar hemodynamic response using NIRS will be the state maker of change in depressive moods of children having depression, similar to that in adults. In the clinical field, pharmacotherapy for children with depression should be considered after psychosocial treatment because of various risks such as activation syndrome and suicidal attempts [8]–[10]. Clinicians have to select drugs for use in depressive children very carefully. Therefore, clinicians can evaluate the efficacy of medication by evaluating frontopolar hemodynamic responses using NIRS as a biomarker before and after pharmacotherapy. NIRS is very convenient and quick to use and it is an innocuous method of examination in children.

However, this study has a number of limitations. The subjects were only 10 children with mild or moderate depression. It is necessary to evaluate more children and their concentration of frontal cortex and depressive mood of children with mild, moderate and severe depression. In this pilot study using two-channel NIRS, the concentration of all frontal cortex of children with depression was not evaluated. It is necessary to evaluate the relationship between the concentration of other parts of the frontal cortex and depressive mood using the multi-channel NIRS. Furthermore, it is important for biomarker of childhood depression that these concentrations were compared with typically development sample and children with depression.

The current study was not a case-control study; it was an observational study and the subjects were children with depression. There is no evidence that the concentration of oxy-Hb of frontopolar in children with depression was significantly decreased compared with that of normal children.

The participants of this study had only mild to moderate depression treated with psychosocial therapy and we focused on how pharmacotherapy would affect hemodynamic response. Furthermore, it was important to evaluate children with severe depression using NIRS and DSRS.

NIRS lacks the precision and depth of fMRI, which can pinpoint changes in blood flow throughout the brain with much greater spatial resolution [7]. However, it is easier to use NIRS to examine children.

This study is the first to evaluate the frontopolar hemodynamic response of children with depression using NIRS. However, NIRS does not show reproducible results in the various clinical settings for children, and there is not much clarity on how to apply these results to mental health. Future studies should be conducted to study depression among children and adolescent using NIRS and other biological examinations.

In conclusion, this study showed that the brain activity assessed by NIRS is a state maker for the depressive mood in children with depression.
